# Cell division cycle 20 promotes tumor progression and predicts poor clinical outcome in childhood and adult adrenocortical carcinoma^☆^

**DOI:** 10.1016/j.jcte.2025.100406

**Published:** 2025-07-02

**Authors:** Jiahong Chen, Peisheng Huang, Yongcheng Shi, Shanshan Mo, Cheng-Ya Hsu, Shumin Fang, Chuanfan Zhong, Le Zhang, Lanting Zuo, Jianming Lu, Weide Zhong, Zhuoya Huang, Zhong Dong

**Affiliations:** a Department of Urology, Huizhou Central People’s Hospital, Huizhou 516001 Guangdong, China; b The First Clinical Medical College, Guangdong Medical University, Zhanjiang 524023 Guangdong, China; c Department of Andrology, Guangzhou First People’s Hospital, Guangzhou Medical University, Guangzhou 510180, China; d Science Research Center, Huizhou Central People’s Hospital, Huizhou 516001 Guangdong, China; e Department of Urology, Zhujiang Hospital, Southern Medical University, Guangzhou 510282 Guangdong, China; f Institute for Integrative Genome Biology, University of California, Riverside 92507 CA, USA; g Guangdong Provincial Key Laboratory of Urology, The First Affiliated Hospital of Guangzhou Medical University. Guangzhou 510000 Guangdong, China; h Department of Pathology, Huizhou Central People’s Hospital, No. 41, Eling North Road, Huizhou 516001 Guangdong, China

**Keywords:** CDC20, ACC, Prognosis, Immunotherapy, Biomarker

## Abstract

**Background:**

Adrenocortical carcinoma (ACC) is an uncommon and highly aggressive tumor with a grim prognosis. Numerous investigations have elucidated a close association between the dysregulated expression of multiple genes within tumors and the initiation as well as progression of neoplasms. These dysregulated genes not only exert pivotal roles in tumorigenesis but also harbor significant potential as prognostic biomarkers.

**Methods:**

This study utilized transcriptomic data from public databases of ACC and normal tissue samples to screen for differentially expressed genes (DEGs). Subsequently, univariate Cox regression and receiver operating characteristic (ROC) curve were employed to identify potential prognostic biomarkers for ACC. Immunohistochemistry and in vitro cell experiments were conducted to validate the expression and potential functions of Cell division cycle 20 (CDC20) in ACC cells. Additionally, we analyzed the relationship between CDC20 and CD8+ T cells, immunotherapy response, somatic mutations, and copy number variations.

**Results:**

CDC20 has emerged as an independent adverse prognostic factor in ACC, with significantly elevated expression levels. In vitro cell experiments have demonstrated that downregulation of CDC20 expression suppresses proliferation and migration of ACC cells. Notably, our study has identified CDC20 expression as most closely associated with TP53 mutation. Additionally, CDC20 expression levels exhibit a negative correlation with infiltration of CD8+ T cells. Patients with low CDC20 expression may show improved response to anti-PD-1 immunotherapy.

**Conclusion:**

CDC20 serves as a reliable and robust biomarker in ACC, playing a crucial role in predicting survival outcomes and assessing immunotherapy response in adult and childhood ACC patients.

## Highlights

• CDC20 overexpression in pediatric/adult adrenocortical carcinomas links to worse prognosis.
• CDC20 silencing inhibits tumor cell proliferation, migration, and invasion in vitro.
• Low CDC20 expression enhances anti-PD-1 immunotherapy sensitivity in patients.

## Introduction

Adrenocortical carcinoma (ACC) is a rare and highly aggressive malignancy, with an annual incidence estimated at 0.5–2 cases per million individuals [1]. ACC can present at any age, but peaks in prevalence are observed in children and individuals aged 40–50 years, with a higher predominance in females [2]. Despite its low frequency, ACC typically carries a dismal prognosis, with a 5-year overall survival rate ranging from 15 % to 44 % [3,4]. Surgical resection remains the cornerstone of curative treatment for early-stage localized tumors; however, the possibility of recurrence persists even after complete excision [5]. In advanced stages of ACC, a multimodal approach combining chemotherapy and local radiotherapy may offer some improvement in prognosis. Nevertheless, the contentious issues persist regarding the toxic side effects stemming from prolonged chemotherapeutic drug administration and the benefits associated with extended survival [6]. Despite recent advancements in the diagnosis and therapeutic management of ACC, the inherent tumor heterogeneity and the limited efficacy of available therapies mean that only a small subset of patients derive notable survival advantages [7].

In the context of rapidly evolving biotechnology, multi-omics methodologies involving genomics, transcriptomics, proteomics, and metabolomics have emerged as indispensable tools in cancer research [8]. Recent breakthroughs have unveiled a plethora of biomarkers crucial in the diagnosis, prognosis assessment, and therapeutic response of tumors. Notably, novel non-invasive biomarkers, such as microRNAs, steroid profiling, circulating tumor cells, and circulating tumor DNAs, have exhibited substantial promise in the diagnostic, prognostic, and surveillance dimensions of ACC [9]. Of note is the increased incidence of childhood ACC in southern Brazil, potentially linked to TP53 mutations [10]. Despite the advancements made by these biomarkers in the diagnostic and prognostic evaluation of ACC, their clinical validity warrants further exploration. Hence, a comprehensive exploration of the correlation between gene expression patterns and clinical outcomes in ACC patients, coupled with the discovery of fresh prognostic markers, holds profound significance.

Cell division cycle 20 (CDC20) serves as a pivotal player in cell cycle regulation. At the culmination of the cell division cycle, CDC20 orchestrates the activation of the anaphase-promoting complex to facilitate chromosomal separation, thereby modulating the cell division process [[11], [12], [13]]. Multiple investigations have elucidated the heightened expression of CDC20 in urothelial bladder cancer [14], non-small cell lung cancer [15], gastric cancer [16], and colorectal cancer [17], associating with unfavorable prognostic implications. Despite the overexpression of CDC20 in various malignancies and its prognostic significance, its potential involvement in ACC remains obscure.

In this study, we utilized transcriptomic data from public databases to perform a differentially expressed gene analysis between ACC and normal tissue samples. We integrated gene expression levels with clinical prognosis data of ACC patients, utilizing univariate Cox regression analysis and receiver operating characteristic (ROC) curve analysis to identify a novel biomarker. Validation of the relationship between CDC20 expression and clinical outcomes in adult and childhood ACC cohorts was performed through Kaplan-Meier survival analysis. Furthermore, we explored the expression of CDC20 protein in ACC through immunohistochemistry (IHC) and investigated the role of CDC20 in ACC cells through in vitro experiments. Additionally, we assessed the biological functions of CDC20 through gene set enrichment analysis (GSEA), immune infiltration analysis associated with immunotherapy, and genomic investigations.

## Materials and methods

### Data collection and preprocessing

Transcriptomic and clinical datasets were acquired from the GEO website (https://www.ncbi.nlm.nih.gov/geo/) comprising four datasets, including two adult ACC cohorts (GSE19750, GSE10927) [18] processed using the “affy” R package (version 1.76.0) [19] with the RMA algorithm for normalization and log2 transformation, and two childhood ACC cohorts (GSE76019, GSE76021) [20]. Adrenocortical adenoma samples were excluded from all four datasets, retaining only ACC and normal tissue samples. Specifically, the dataset GSE19750 comprises 44 ACC samples and 4 normal adrenal samples. GSE10927 includes 33 ACC samples and 10 normal adrenal samples. GSE76019 contains 34 ACC samples, and GSE76021 includes 19 ACC samples. The two merged datasets (GSE19750-10927, GSE76019-GSE76021) were combined from the two adult ACC cohorts and two childhood ACC cohorts, respectively, and batch correction was performed using the “sva” R package (version 3.46.0) [21]. Batch effects within the GSE19750 dataset were also corrected using the same method. Expression data from RNA-seq (FPKM) and associated clinical information for 79 TCGA-ACC samples were retrieved from the UCSC Xena website (https://xenabrowser.net/Datapages/). Somatic mutation data for ACC were downloaded from the TCGA GDC website (https://portal.gdc.cancer.gov/), along with copy number variation (CNV) data processed using the GISTIC2.0 algorithm. The sample information of the above-mentioned datasets has been compiled in Table S1.

### Differentially expressed gene analysis

The GSE19750-10927 dataset was subjected to differentially expressed gene analysis using the “limma” R package (version 3.54.0) [22], selecting differentially expressed genes (DEGs) based on |log2 FC| > 2 and adj. P < 0.05 criteria.

### Functional enrichment analysis

DEGs were categorized into upregulated and downregulated genes based on expression differences between ACC and normal samples. This was followed by Gene Ontology (GO) enrichment analysis, facilitated by the “clusterProfiler” R package (version 4.6.0) [23], aimed at delineating the functional roles of DEGs within cellular components, molecular functions, and biological processes.

### Univariate Cox regression analysis and ROC curve analysis for biomarker selection

Univariate Cox regression analysis was implemented employing the “survival” R package (version 3.4-0) [24] to scrutinize the association between gene expression patterns and patient survival outcomes. Genes exhibiting a Hazard Ratio (HR) > 1 and P-value < 0.05 were regarded as adverse prognostic factors. Subsequently, ROC curve analysis, employing the “timeROC” R package (version 0.4) [25], was conducted to evaluate the predictive efficacy of genes concerning median survival time, with those demonstrating an area under curve (AUC) > 0.7 deemed to possess substantial predictive utility.

### Kaplan-Meier survival analysis and multivariable Cox regression analysis for gene prognostic evaluation

CDC20 expression levels were dichotomized into high and low expression cohorts utilizing the optimal threshold identified by the “survminer” R package. Subsequent Kaplan-Meier survival analysis and log-rank tests, executed via the “survival” R package, were utilized to examine the influence of CDC20 expression on overall survival (OS), event-free survival (EFS), and progression-free interval (PFI) within ACC cohorts. Moreover, multivariable Cox regression analysis was undertaken to evaluate the predictive capacity of CDC20, adjusting for variables including gender, age, and tumor stage.

### Gene set enrichment analysis

Analysis utilizing two datasets, TCGA and GSE76019, was undertaken to elucidate the biological functions of CDC20 in both adult and childhood ACC. Spearman correlation was utilized to evaluate the association between CDC20 expression and coding genes, with genes demonstrating statistical significance at P < 0.05 based on correlation coefficients being subjected to further investigation. Furthermore, GSEA pertaining to GO terms was performed using the “clusterProfiler” R package to provide additional insights into the biological functions influenced by CDC20.

### Immunohistochemistry

We obtained 11 tissue samples from Huizhou Central People’s Hospital, comprising 6 cases of ACC and 5 cases of adrenocortical adenoma. Ethical approval for this study was granted by the ethics committee under the number KYLL2023106. Following the established protocol from our previous research [26], the methodology is summarized as follows. The anti-CDC20 antibody (YT0791, Immunoway) was utilized for this IHC. The samples were initially fixed in 4 % paraformaldehyde and subsequently embedded in paraffin. Tissue sections, each 4 µm in thickness, were treated with a 1 % H_2_O_2_ solution to eliminate endogenous peroxidase activity, followed by blocking with non-immune goat serum to reduce non-specific binding. The sections were then incubated with primary antibodies overnight at 4 °C, and subsequently with biotinylated secondary antibodies for 30 min at room temperature. The scoring of IHC staining intensity covered a scale ranging from 0 to 3: 0 designated no positive staining (negative), 1 indicated faint yellow (weak positive), 2 represented light brown (positive), and 3 signified dark brown (strong positive) staining. Similarly, the percentage of cells demonstrating positive staining was graded on a scale from 1 to 4: 1 for ≤25 %, 2 for 26 %–50 %, 3 for 51 %–75 %, and 4 for >75 %. The cumulative score was computed by multiplying the intensity and distribution score.

### Cell lines and cell transfection

In accordance with the protocol established in our previous studies [26], a concise overview is presented below. This investigation utilized two human ACC cell lines: SW-13 and NCI-H295R. The cells were cultured in DMEM (BC-M-005, Bio-channel) and DMEM/F12 medium (BC-M-002, BioChannel), both supplemented with 10 % fetal bovine serum (BC-SE-FBS07, Bio-Channel), and maintained in a humidified incubator at 37 °C with 5 % CO_2_. Following the manufacturer’s guidelines, negative control (NC) and CDC20 siRNA (Genepharma, Guangzhou, China) were transfected into the ACC cells using GP-transfect-Mate (GemPharmatech, Suzhou, China). The plates were incubated for 48 h prior to harvesting total protein for Western blot analysis. The sequences of the three siRNAs are as follows:

siRNA#1: 5′-GGUUAUCAGAACAGACUGA-3′,
siRNA#2: 5′-CAGACAUUCACCCAGCAUCAA-3′,
siRNA#3: 5′-GCACAGAACCAGCUAGUUA-3′.

### Western blotting

Cells derived from the two ACC cell lines were lysed using RIPA buffer supplemented with protease inhibitors. Subsequently, the extracted proteins underwent separation via SDS-PAGE gel electrophoresis, followed by transfer onto PVDF membranes and blocking with 5 % skim milk. The membranes were then subjected to incubation with a primary anti-CDC20 antibody (YT0791, Immunoway) at a dilution of 1:2000, after which they were probed with a secondary antibody. Following three washes with PBST, each lasting 10 min, the membranes were exposed. GAPDH served as the internal reference control. Finally, utilizing Image J software, the intensities of protein bands were quantified.

### Colony formation assay

Following transfection, cells from both cell lines were seeded at a density of 1000 cells per well in 6-well plates and cultured in a 37 °C, 5 % CO_2_ cell culture incubator for a duration of 2 weeks. Subsequently, the cells were rinsed twice with cold PBS, fixed with 4 % paraformaldehyde for 15 min, and stained with 1 % crystal violet solution at room temperature for 20 min. Images were captured, and colonies were enumerated.

### Transwell assay

To assess the migratory capability, a transwell assay was employed. Approximately 4 × 10^4^ transfected cells were plated in the upper chamber with serum-free medium, while the lower chamber contained complete medium. After a 48-hour incubation under standard culture conditions, the cells were washed with PBS, fixed in paraformaldehyde, and stained with 0.1 % crystal violet. The stained cells were then examined microscopically and quantified.

### Comprehensive analysis of immune infiltration and immunotherapy.

To explore the tumor microenvironment (TME) and immunotherapy outcomes in adult and childhood ACC, we utilized the “IOBR” package (version 0.99.9) [27] to calculate CD8+ T cell infiltration abundance using TIMER and Xcell algorithms for the TCGA and GSE76019 cohorts. We analyzed the correlation between CD8+ T cell levels and CDC20 expression. We evaluated patient response to immunotherapy utilizing the tumor immune dysfunction and exclusion (TIDE) [28] web tool for the TCGA and GSE76019 cohorts, examining the correlation between CDC20 expression levels and TIDE scores. Subclass mapping (Submap) [29], is a commonly utilized unsupervised clustering algorithm, primarily employed to discover shared subtypes across different cohorts. In this study, the Submap was employed to assess the response of patients with varying CDC20 expression levels in the TCGA and GSE76019 cohorts to anti-PD-1 and anti-CTLA-4 immunotherapy. An adj P < 0.05 was deemed significant. Transcriptional data and clinical data from two melanoma cohorts [30,31], one renal cell carcinoma cohort [32], and one glioblastoma cohort [33] from the TIGER database, where patients received anti-PD-1 immunotherapy, were downloaded for Kaplan-Meier survival analysis.

### Mutation and copy number variation in ACC

The somatic mutation dataset was analyzed employing the “maftools” package (version 2.14.0) [34] to assess the mutation frequency of each gene. Waterfall plots were created to visualize the top 20 genes exhibiting high mutation frequencies, and the discrepancy in mutation frequencies of these 20 genes between subgroups characterized by high and low CDC20 expression levels was examined. Additionally, we used the “ComplexHeatmap” package (version 2.14.0) [35] to visualize the top 10 high-frequency amplified chromosome segments and top 10 high-frequency deleted chromosome segments, analyzing the relationship between CDC20 expression levels and CNV of these segments.

### Statistical analysis

For this investigation, R software version 4.2.1 was utilized for data processing and visualization. Selected plots were generated using Sangerbox (https://vip.sangerbox.com/) [36]. Spearman correlation analysis was conducted. Kaplan-Meier survival analysis was utilized to compare survival outcomes between high and low expression subgroups, with significance determined by log-rank tests. The predictive accuracy of gene models was determined using AUC values. Variations between two data groups were analyzed using independent sample Wilcoxon tests or t-tests, and Fisher’s exact test was applied to examine the relationship between mutation data and CDC20 expression levels. Two-tailed P-values were calculated, with significance levels denoted as *P < 0.05, **P < 0.01, ***P < 0.001, ****P < 0.0001.

## Results

### Batch correction and screening of DEGs

**Fig. 1:**
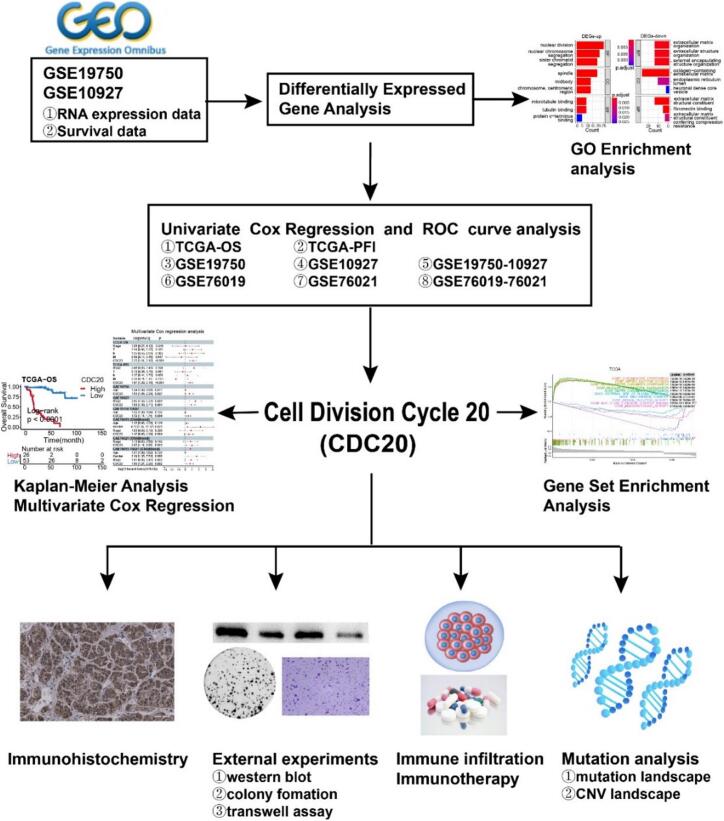
Workflow of this study.

**Fig. 2:**
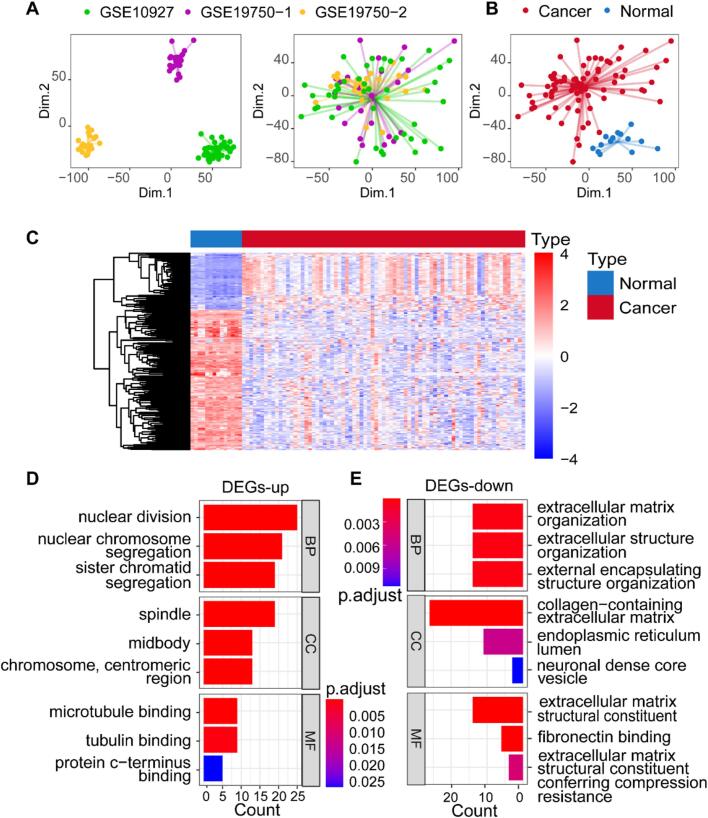
Differentially expressed genes (DEGs) between ACC and normal tissues. (A-B) Batch correction of expression data from GSE10927 and GSE19750 to generate the GSE10927-19750 cohort. (C) Heatmap of DEGs between ACC and normal tissues. (D) Enrichment analysis results of upregulated genes using Gene Ontology (GO). (E) Enrichment analysis results of downregulated genes using GO.

The flow chart of this study is shown in Fig. 1. To identify DEGs between ACC and normal tissues, we downloaded expression profile data from two adult ACC datasets (GSE10927, GSE19750) and two childhood ACC datasets (GSE76019, GSE76021) from the GEO website. We merged the two adult ACC datasets after batch correction into the GSE10927-19750 cohort (Fig. 2A-B) and batch-corrected the two childhood ACC datasets into the GSE76019-76021 cohort (Fig. S1A-B). Internal batch effects in the GSE19750 dataset were removed. We performed differentially expressed gene analysis on the GSE10927-19750 cohort and identified 233 DEGs based on the criteria of |log2 FC| > 2 and adjusted P-value < 0.05 (Table S2). These DEGs showed significant expression differences between ACC and normal tissues (Fig. 2C).

### Functional enrichment analysis of DEGs

In order to elucidate the roles played by DEGs in ACC, we conducted separate functional enrichment analyses for both upregulated and downregulated DEGs. The top 3 GO terms per ontology were visualized based on adjusted P-values. Upregulated DEGs were found to be enriched in GO terms related to sister chromatid segregation, spindle, midbody, and microtubule binding, while downregulated DEGs showed enrichment in GO terms associated with extracellular matrix organization and fibronectin binding (Fig. 2D-E, Table S3). These findings suggest that these genes may facilitate the progression of ACC by influencing processes such as cell division and extracellular matrix structure.

### Identification and validation of prognostic genes

**Fig. 3:**
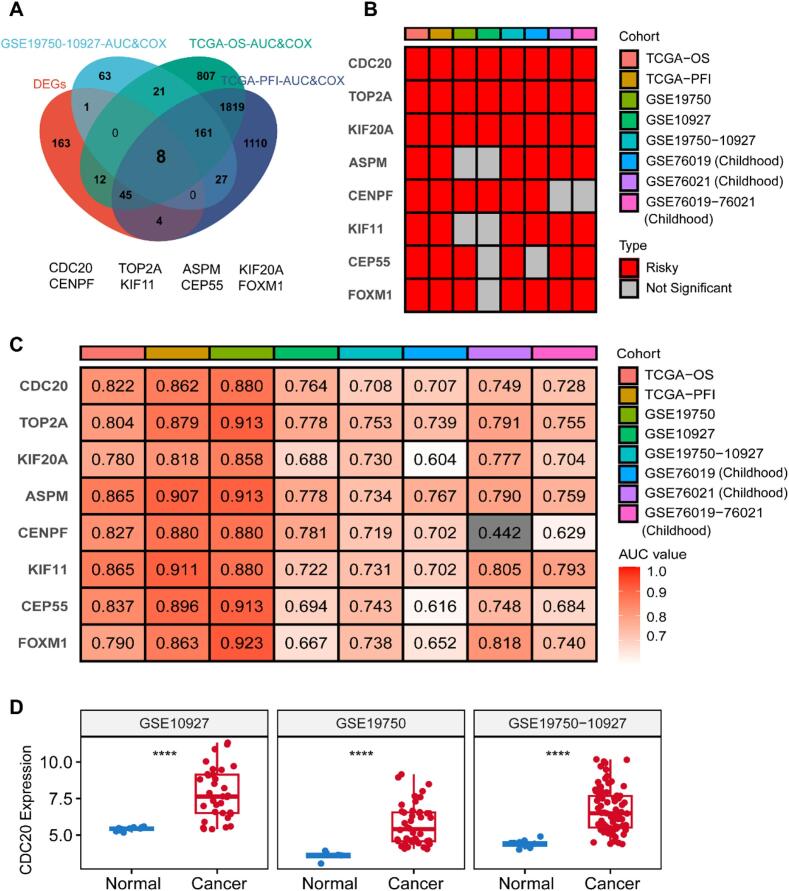
Identification of core gene CDC20 in ACC. (A) By integrating DEGs, genes with AUC values > 0.7, HR > 1, and P-values < 0.05 in the GSE10927-19750 dataset, and genes with AUC values > 0.7, HR > 1, and P-values < 0.05 in TCGA datasets for overall survival (OS), progression-free interval (PFI), we identified 8 core genes. (B) Univariate Cox regression analysis of the 8 core genes across all cohorts. (C) AUC values of the 8 core genes across all cohorts. (D) Expression levels of CDC20 between ACC and normal tissues in GSE10927, GSE19750, and GSE10927-19750 cohorts. *P < 0.05, **P < 0.01, ***P < 0.001, ****P < 0.0001.

As illustrated in the Venn diagram of Fig. 3A, we identified eight prognostic-related genes (CDC20, TOP2A, ASPM, CENPF, KIF11, CEP55, KIF20A, and FOXM1) by integrating DEGs, genes with AUC values > 0.7, HR > 1, and P-values < 0.05 in the GSE10927-19750 dataset, and genes with AUC values > 0.7, HR > 1, and P-values < 0.05 in TCGA datasets for OS and PFI. Subsequently, we evaluated the univariate Cox regression analysis results and AUC values of these 8 genes across all cohorts (Fig. 3B-C). Genes with HR > 1 and P-value < 0.05 in the univariate Cox regression analysis and AUC > 0.7 in ROC curve analysis were considered pivotal. Thus, we narrowed down to CDC20 and TOP2A as central genes. Nevertheless, there has been no documentation regarding the potential implications of CDC20 in ACC. Hence, our study seeks to elucidate the role of CDC20 as a pivotal gene in ACC. Furthermore, in the GSE10927-19750, GSE10927, and GSE19750 cohorts, CDC20 exhibited significantly elevated expression levels in tumor tissues relative to normal tissues (Fig. 3D).

**Fig. 4:**
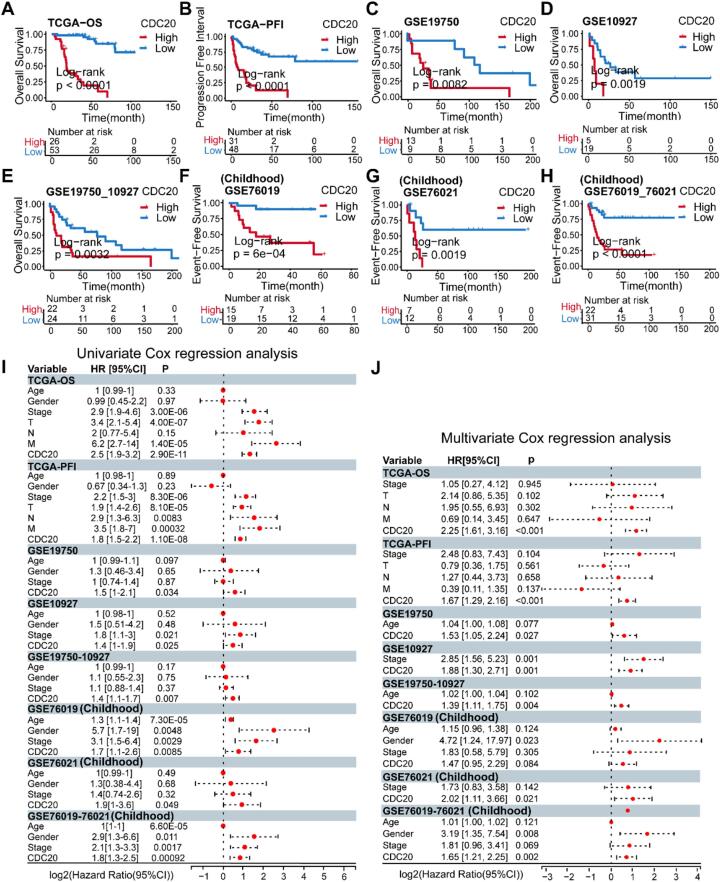
Validation of CDC20 as an independent prognostic factor for ACC. (A–H) Kaplan-Meier survival analysis of CDC20 across all cohorts. (I) Univariate Cox regression analysis of CDC20, age, gender, and tumor stage across all cohorts. (J) Multivariate Cox regression analysis of CDC20 across all cohorts.

To validate the prognostic significance of CDC20 in ACC, we stratified CDC20 into high and low expression cohorts based on the optimal cutoff value and conducted Kaplan-Meier survival analysis coupled with log-rank tests. Across all cohorts, the high CDC20 expression cohort displayed inferior prognosis compared to its counterpart (Fig. 4A–H). Both univariate and multivariate Cox regression analyses indicated that, with the exception of the GSE76019 cohort, CDC20 not only significantly predicted OS, EFS, and PFI but also retained its status as an independent prognostic factor for ACC even after adjusting for clinical covariates such as patient age, gender, and tumor stage (Fig. 4I–J). In summary, CDC20 emerges as a dependable prognostic biomarker for both adult and childhood ACC patients.

### Biological function analysis of CDC20

**Fig. 5:**
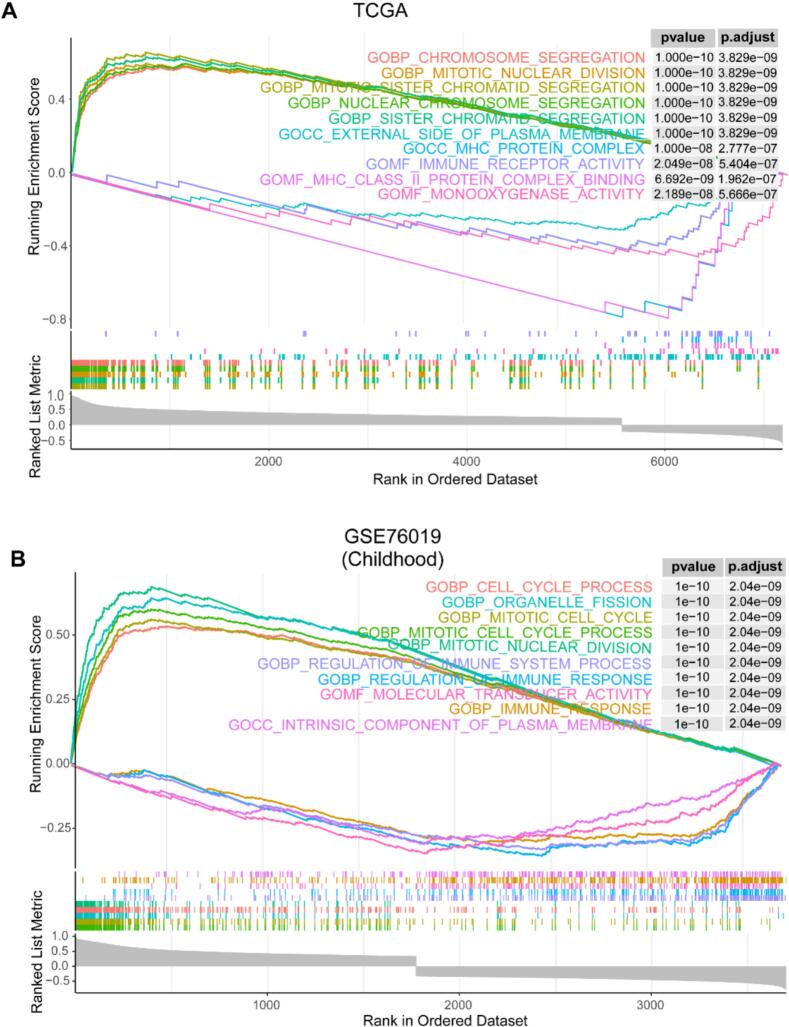
The gene set enrichment analysis (GSEA) results for the TCGA and GSE76019 datasets. (A) GSEA results of CDC20-related GO terms in TCGA dataset. (B) GSEA results of CDC20-related GO terms in GSE76019 dataset.

We further investigated the potential biological functions of CDC20 in adult and childhood ACC by conducting GSEA based on the correlation of CDC20 with other coding genes. We visualized the top 5 GO terms ranked by normalized enrichment score (NES) in two datasets. As shown in Fig. 5A and Table S4, in the adult TCGA dataset, genes significantly positively correlated with CDC20 expression enriched in GO terms such as sister chromatid segregation, chromosome segregation, etc. Conversely, genes significantly negatively correlated with CDC20 expression enriched in GO terms including monooxygenase activity, MHC protein complex, immune receptor activity. In the GSE76019 dataset, CDC20 was found to be involved in activating GO terms such as mitotic cell cycle process, organelle fission, cell cycle process, while inhibiting GO terms like regulation of immune system process, regulation of immune response, immune response (Fig. 5B, Table S5). These findings suggest that CDC20 may promote progression of adult and childhood ACC by activating cell division processes and suppressing immune response pathways.

### IHC staining validation of CDC20 protein overexpression in ACC

**Fig. 6:**
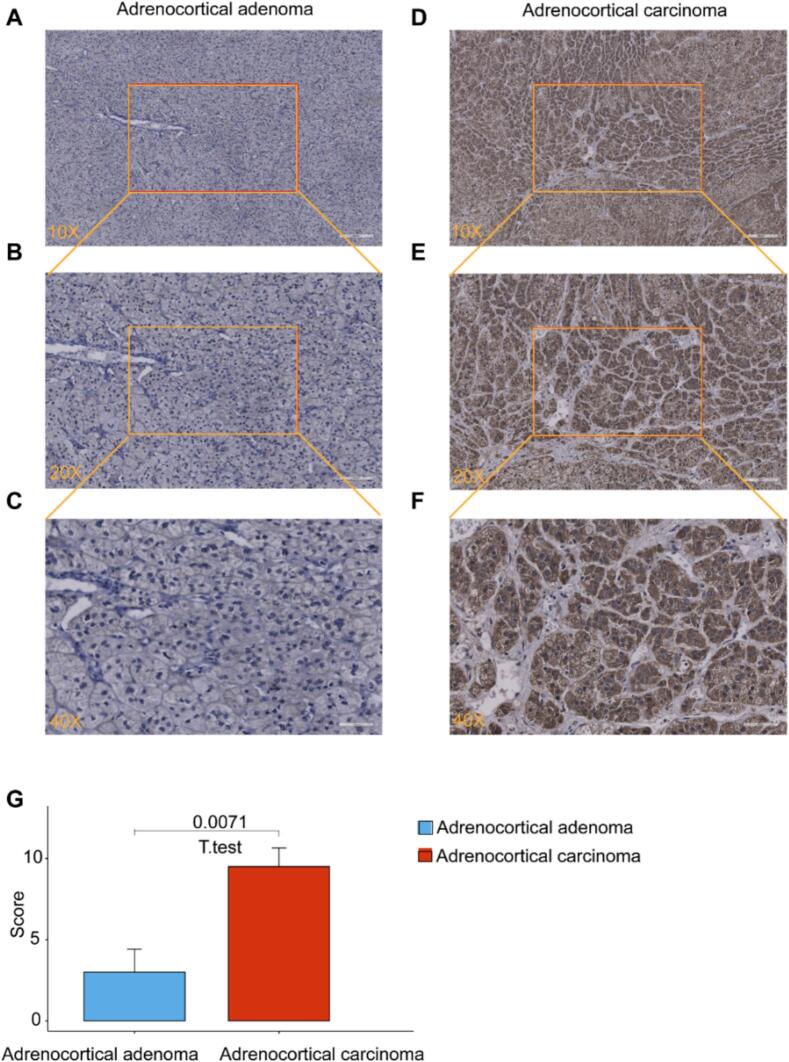
Immunohistochemistry (IHC) staining results of CDC20 in ACC and adrenocortical adenoma. (A–C) Microscopic images (10×, 20×, 40×) of CDC20 IHC staining in adrenocortical adenoma. (D–F) Microscopic images (10×, 20×, 40×) of CDC20 IHC staining in ACC. (G) IHC scoring comparison between ACC (n = 6) and adrenocortical adenoma (n = 5) tissues.

In order to validate the hypothesis of increased CDC20 expression in ACC tissues over non-cancerous adrenal tissues, we applied IHC staining to evaluate both the expression level and subcellular localization of CDC20 in 6 cases of ACC and 5 cases of adrenocortical adenoma. The findings revealed pronounced CDC20 expression, primarily in the cytoplasm of ACC cells (Fig. 6D–F), whereas adrenocortical adenoma tissues exhibited weak expression or were completely negative for CDC20 (Fig. 6A–C). The IHC scores used on all 11 tissue samples showed significantly higher scores in the ACC group compared to the adrenocortical adenoma group (Fig. 6G).

### CDC20 promotes proliferation and migration of ACC

**Fig. 7:**
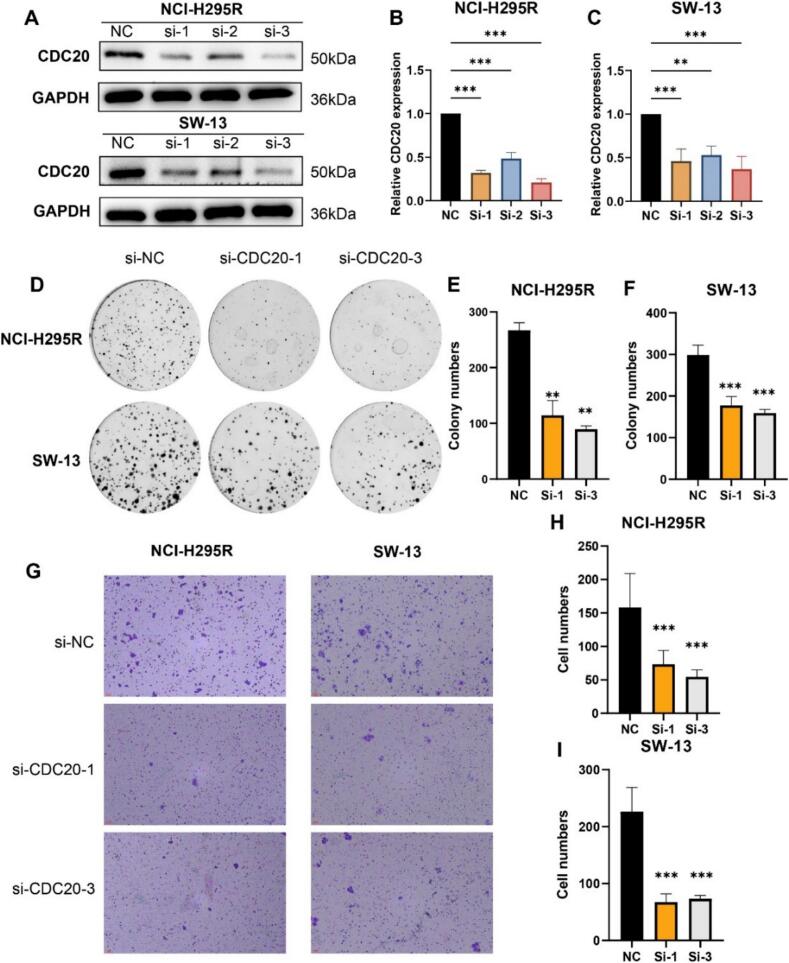
Cell experiments. (A) Western blotting to assess the impact of 3 siRNAs on CDC20 expression in ACC cells. (B–C) Quantitative analysis of CDC20 expression after CDC20 gene knockdown in NCI-H295R (B) and SW-13 (C) cell lines. (D) Colony formation assay results in NCI-H295R and SW-13 cell lines. (E–F) Quantification of proliferating cells in NCI-H295R (E) and SW-13 (F) cell lines. (G) Transwell assay results in NCI-H295R and SW-13 cell lines. (H–I) Quantification of migrating cells in NCI-H295R (H) and SW-13 (I) cell lines. *P < 0.05, **P < 0.01, ***P < 0.001, ns indicates no statistical significance.

We utilized the NCI-H295R and SW-13 cell lines to validate the role of CDC20 in ACC. Initially, we designed three specific siRNAs to suppress CDC20 expression in the NCI-H295R and SW-13 cell lines. We found that siRNA1 and siRNA3 significantly reduced CDC20 expression (Fig. 7A–C). Subsequently, using siRNA1 and siRNA3 in colony formation assay and transwell assay experiments, we downregulated CDC20 expression in the NCI-H295R and SW-13 cell lines. The colony formation assay indicated that knocking down CDC20 significantly inhibited the proliferation capacity of NCI-H295R and SW-13 cells (Fig. 7D–F). The transwell assay results demonstrated that knocking down CDC20 significantly suppressed the migration capabilities of NCI-H295R and SW-13 cells (Fig. 7G–I). In summary, downregulation of CDC20 expression attenuates the proliferative and migratory capacities of ACC cells. Hence, CDC20 facilitates the malignant progression of ACC.

### Analysis of CDC20 with CD8+ T cells and immunotherapy response

**Fig. 8:**
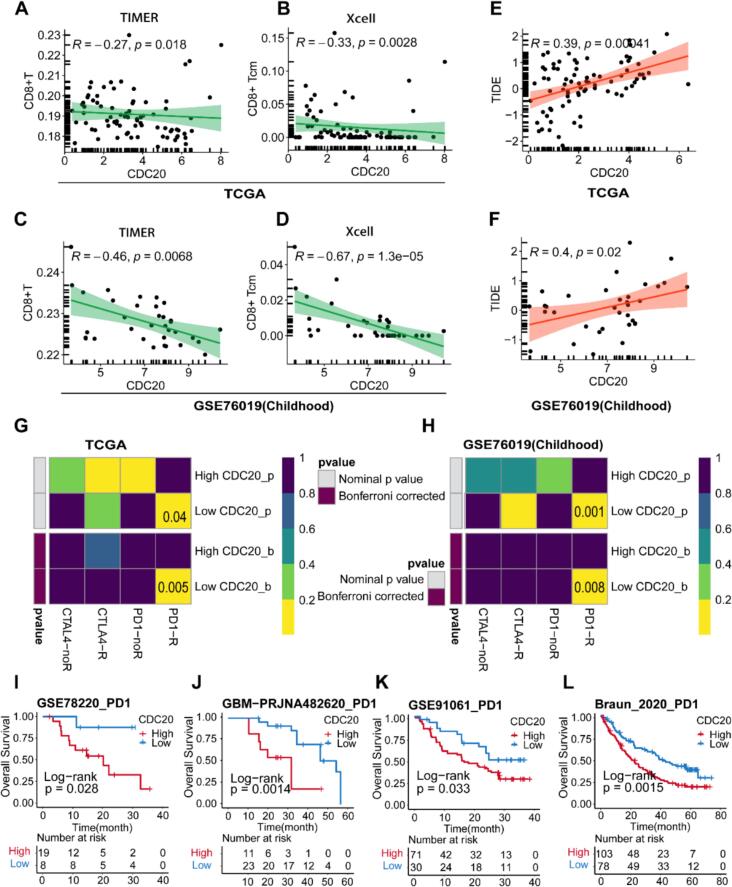
Immune infiltration and immunotherapy. (A–D) Correlation analysis between CDC20 expression and CD8+ T cell infiltration levels using TIMER and Xcell algorithms in TCGA and GSE76019 datasets. (E–F) Assessment of tumor immune dysfunction and exclusion (TIDE) score correlation with CDC20 expression levels in TCGA and GSE76019 datasets. (G–H) Subclass mapping (Submap) algorithm evaluation of expression similarity between two CDC20 expression subgroups and different immunotherapy response patients in TCGA and GSE76019 datasets. (I–L) Kaplan-Meier survival analysis of high and low CDC20 expression subgroups among patients receiving anti-PD-1 therapy in four cohorts.

To explore the relationship between CDC20 expression and CD8+ T cell infiltration within the TME in adult and childhood ACC, we employed TIMER and Xcell algorithms to quantify the relative abundance of CD8+ T cell infiltration. Analyzing both the TCGA and GSE76019 cohorts, we observed a negative correlation between CDC20 expression levels and the relative abundance of CD8+ T cells (Fig. 8A–D). Furthermore, we assessed the immunotherapy outcomes in both adult and childhood ACC using the TIDE algorithm to predict immune checkpoint blockade (ICB) responsiveness based on individual patient data from these cohorts. As indicated in Fig. 8E–F, there was a positive correlation between CDC20 expression levels and TIDE scores, suggesting that patients with higher CDC20 expression may exhibit poorer responses to ICB therapy. The Submap algorithm was utilized to investigate the differential responses to anti-CTLA-4 and anti-PD-1 targeted immunotherapy among ACC patients with high versus low CDC20 expression. The results indicated a more favorable response to anti-PD-1 therapy in patients with lower CDC20 expression (Fig. 8G–H). Further analysis of the survival prognosis among different CDC20 expression subgroups in four cohorts treated with anti-PD-1 immunotherapy demonstrated that patients with high CDC20 expression had worse OS (Fig. 8I–L).

### CDC20-associated gene mutations and copy number variations

**Fig. 9:**
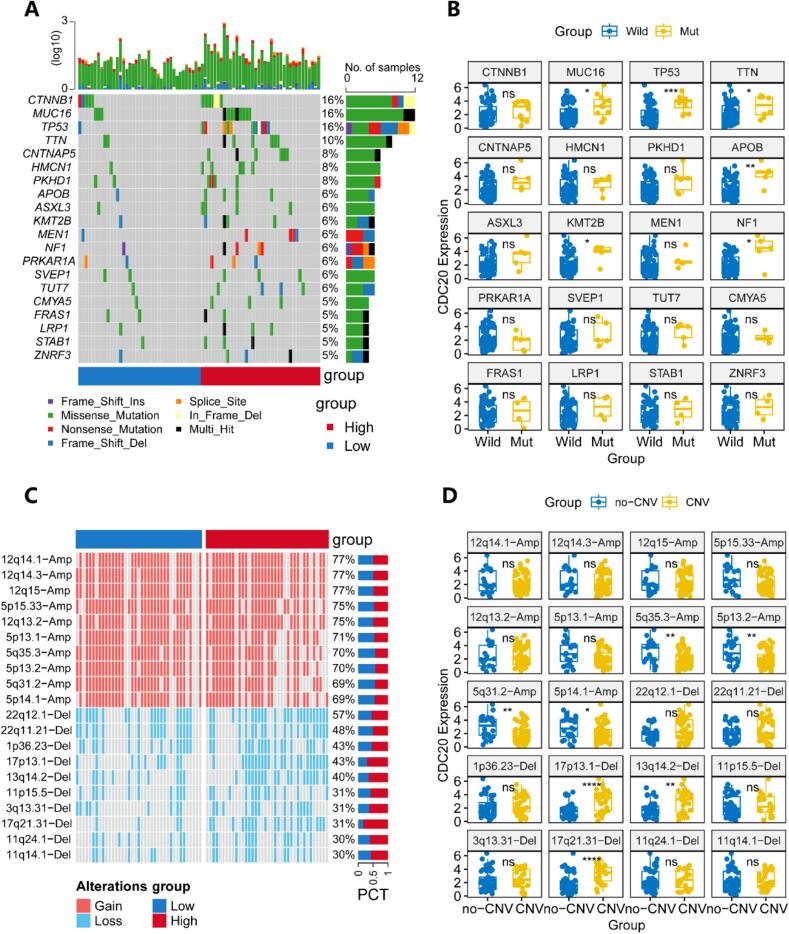
Analysis of CDC20-related mutation and copy number variation (CNV). (A) Landscape of top 20 frequent mutated genes. (B) Differential expression of CDC20 between wild-type and mutated groups among the top 20 frequent mutated genes. (C) Landscape of top 10 chromosomal amplifications and top 10 chromosomal deletions. (D) Differential expression of CDC20 between variant and non-variant groups among the 10 chromosomal amplifications and top 10 chromosomal deletions. *P < 0.05, **P < 0.01, ***P < 0.001, ****P < 0.0001, ns indicates no statistical significance.

To explore the potential relationship between specific genomic mutations and CDC20 expression, we profiled the top 20 genes with the highest mutation rates as shown in Fig. 9A. Subsequent analysis revealed that mutations in MUC16, TP53, TTN, APOB, KMT2B and NF1 were significantly associated with increased CDC20 expression (Fig. 9B). Particularly noteworthy, TP53 and CNTNAP5 mutations demonstrated a marked prevalence in the cohort with high CDC20 expression (Fig. S2A). Similarly, we analyzed the CNV status of the top 10 high-frequency amplified chromosomal segments and the top 10 high-frequency deleted chromosomal segments (Fig. 9C). We observed that the expression levels of CDC20 in the amplification groups of 5q35.3, 5p13.2, 5q31.2, and 5p14.1 were significantly lower than those in the non-variant groups, while the expression levels of CDC20 in the deletion groups of 17p13.1, 13q14.2, and 17q21.31 were significantly higher than those in the non-variant groups (Fig. 9D). By examining the frequency of chromosomal segment variations between CDC20 high and low expression subgroups, we found that only the deletion frequency of chromosomal segments like 17p13.1, 13q14.2, and 17q21.31 was significantly higher in the CDC20 high expression group compared to the CDC20 low expression group (Fig. S2B).

## Discussion

ACC is extremely rare, highly malignant, and lacks effective treatment strategies [2,37]. Despite some progress with new treatment approaches over the past decades, the survival rates of ACC patients have not significantly improved [38]. Given the poor prognosis of ACC patients and the lack of effective treatment options, exploring a new biomarker to assess ACC prognosis is of paramount importance.

Through differentially expressed gene analysis, we identified DEGs between tumors and normal tissues. Subsequently, from the results of GO enrichment analysis, we found that these DEGs are mainly enriched in pathways such as sister chromatid segregation, centrosome, microtubule binding, extracellular matrix organization, and fibroblast growth factor binding. Previous studies have shown that centrosomes and microtubules play crucial roles in cellular processes such as cell division, intracellular transport, and cell migration [[39], [40], [41]]. The extracellular matrix participates in cell signaling and tissue remodeling processes, undergoing extensive remodeling under pathological conditions, and is a key participant driving disease progression [42]. These findings collectively imply a close correlation between these pathways and ACC progression. Notably, CDC20, a cell division cycle 20 homolog, which is primarily involved in cell cycle regulation, demonstrated abnormal expression patterns linked to heightened cell proliferation and tumorigenesis [43,44]. Consistent with literature on various cancers including hepatocellular Carcinoma [45], renal clear cell carcinoma [46], breast cancer [47], and pancreatic ductal adenocarcinoma [48], our study demonstrated that CDC20 is highly expressed in ACC and is associated with poor prognosis. Furthermore, GSEA revealed that GO terms positively correlated with CDC20 expression, including sister chromatid segregation and chromosome segregation, were significantly enriched. This suggests that the CDC20 may promote cell proliferation and division by activating these pathways. Consistently, our in vitro cell experiments demonstrated that CDC20 promotes proliferation and migration of ACC cells. Conversely, GO terms negatively correlated with CDC20 expression, such as regulation of immune system process, regulation of immune response, and immune response. This indicates that CDC20 may inhibit immune response processes, thereby leading to poor prognosis in ACC patients.

Furthermore, our investigation delved into the association between CDC20 and immune infiltration as well as immunotherapy response. Immune infiltration within the TME plays a pivotal role in tumor progression and influences the clinical outcomes of cancer patients [49]. Notably, cytotoxic CD8+ T cells represent a primary subset of immune cells tasked with tumor control and clearance, exerting critical functions in anti-tumor immune processes. Following activation by immunogenic signals, CD8+ T cells migrate to tumor sites to elicit robust immune responses [50]. Prior research has indicated that CDC20 downregulation can bolster an anti-tumor immune response of prostate cancer by facilitating CD8 lymphocyte infiltration dependent on GSDME [51]. In our investigation, we observed a negative correlation between CDC20 expression and CD8+ T cell infiltration abundance across adult and childhood ACC cohorts, suggesting a plausible link between CD8+ T cell exhaustion and elevated CDC20 expression, thereby contributing to compromised immune responses.

ICB therapy is a treatment modality that enhances tumor immune responses by modulating T cell activity, with CD8+ T cells being the main effector cells mediating ICB therapy [52]. Subsequently, we further predicted the response of ACC patients to ICB therapy using TIDE and Submap algorithms. Our findings indicate that ACC patients with reduced CDC20 expression exhibit enhanced sensitivity to ICB therapy. Particularly for anti-PD-1 immunotherapy, patients with low CDC20 expression responded better than those with high CDC20 expression. Furthermore, we found that among patients with melanoma, renal cell carcinoma, and glioblastoma who received anti-PD-1 immunotherapy, those with high CDC20 expression had poorer clinical outcomes. In summary, low CDC20 expression and higher CD8+ T cell infiltration along with upregulated PD-1 expression may benefit ACC patients in immunotherapy, providing important support for evaluating immunotherapy in ACC patients.

Extensive research has established a significant correlation between genetic aberrations and the etiology and development of malignancies. These genetic mutations, encompassing both inherited and somatic variations, are crucial in the carcinogenesis process, facilitating the transformation of healthy cells into malignant counterparts. Such insights highlight the critical role of genomic studies in uncovering the molecular mechanisms underlying cancer and in paving the way for the development of targeted cancer therapies. Through our analysis of CDC20 and ACC mutation patterns, we discovered a close correlation between TP53 mutations and deletions at 17p13.1, 13q14.2, and 17q21.31 with CDC20 expression. The TP53 gene, a cornerstone in tumor suppression, is frequently altered across various human malignancies [53,54]. In southern Brazil, the high incidence of childhood ACC is associated with TP53 mutations [10]. Research by Zhao et al. in hepatocellular carcinoma indicates that wild-type p53 suppresses tumor growth by downregulating CDC20. In tumor cells with mutated p53, this regulatory mechanism is impaired, leading to cell cycle arrest at the G2/M phase and reduced apoptosis-inducing capabilities [55]. Studies have shown that deletions at 1p, 21, 11q, 17p, 22p, 22q1, and amplifications at 4q, 4p16, 5p15, 5q12-13, 5q32-qter, 9q34, 12q13, 19p are associated with the occurrence and development of ACC [56]. Patients with chronic lymphocytic leukemia with 17p13.1 deletion have poor treatment responses [57]. Overexpression of miR-497 located in the chromosome region 17p13.1 downregulates EIF4E expression, inhibiting cell proliferation and inducing cell cycle arrest [58]. Deletion of 13q14.2 is associated with chemotherapy response in breast cancer [59], and 17q21.31 deletion is associated with prostate cancer progression [60]. Therefore, we speculate that TP53 mutations and deletions at 17p13.1, 13q14.2, and 17q21.31 may be correlated with CDC20 expression, thereby promoting ACC progression. The discovery of these molecular targets provides a reference for targeted therapy for ACC.

In summary, through public data analysis, we identified a novel prognostic marker for ACC that can predict the survival prognosis of adult and childhood ACC patients and provide a reference for evaluating immunotherapy for ACC patients. However, the molecular mechanisms of CDC20 in the occurrence and progression of ACC are still unclear. In future studies, we will further investigate this. Additionally, our study has certain limitations. We collected a limited number of samples and lack further experimental validation of our results.

## Conclusion

CDC20 serves as a reliable and robust biomarker in ACC, playing a crucial role in predicting survival outcomes and assessing immunotherapy response in adult and childhood ACC patients.

## CRediT authorship contribution statement

**Jiahong Chen:** Writing – original draft, Methodology, Data curation, Conceptualization. **Peisheng Huang:** Writing – original draft, Visualization, Software, Data curation. **Yongcheng Shi:** Writing – original draft, Visualization, Investigation, Data curation. **Shanshan Mo:** Visualization, Validation. **Cheng-Ya Hsu:** Visualization, Validation. **Shumin Fang:** Resources, Project administration, Conceptualization. **Chuanfan Zhong:** Supervision, Formal analysis. **Le Zhang:** Methodology, Investigation. **Lanting Zuo:** Software, Formal analysis. **Jianming Lu:** Supervision, Methodology. **Weide Zhong:** Methodology, Conceptualization. **Zhuoya Huang:** Writing – review & editing, Validation, Supervision, Conceptualization. **Zhong Dong:** Writing – review & editing, Supervision, Project administration, Methodology, Investigation.

## Funding

This work was supported by the Huizhou Science and Technology Bureau – Medical and Health Project (Self-raised Key Project) (Grant number 2022CZ010004), and the Regional Joint Fund—Regional Cultivation Project (Grant number 2023A1515140040).

## Declaration of competing interest

The authors declare that they have no known competing financial interests or personal relationships that could have appeared to influence the work reported in this paper.

## Data Availability

The public data used in this study have been described in the Materials and Methods.

## References

[1] Kerkhofs T.M., Verhoeven R.H., van der Zwan J.M., Dieleman J., Kerstens M.N., Links T.P. (2013). Adrenocortical carcinoma: a population-based study on incidence and survival in the Netherlands since 1993. Eur J Cancer.

[2] Berruti A., Baudin E., Gelderblom H., Haak H.R., Porpiglia F., Fassnacht M. (2012). Adrenal cancer: ESMO Clinical Practice Guidelines for diagnosis, treatment and follow-up. Ann Oncol.

[3] Schulick R.D., Brennan M.F. (1999). Long-term survival after complete resection and repeat resection in patients with adrenocortical carcinoma. Ann Surg Oncol.

[4] Icard P., Goudet P., Charpenay C., Andreassian B., Carnaille B., Chapuis Y. (2001). Adrenocortical carcinomas: surgical trends and results of a 253-patient series from the French Association of Endocrine Surgeons study group. World J Surg.

[5] Fassnacht M., Dekkers O.M., Else T., Baudin E., Berruti A., de Krijger R. (2018). European Society of Endocrinology Clinical Practice Guidelines on the management of adrenocortical carcinoma in adults, in collaboration with the European Network for the Study of Adrenal Tumors. Eur J Endocrinol.

[6] Kerkhofs T.M., Ettaieb M.H., Hermsen I.G., Haak H.R. (2015). Developing treatment for adrenocortical carcinoma. Endocr Relat Cancer.

[7] Kiesewetter B., Riss P., Scheuba C., Mazal P., Kretschmer-Chott E., Haug A. (2021). Management of adrenocortical carcinoma: are we making progress?. Ther Adv Med Oncol.

[8] Cheng J., He Z., Liu Y., Jing J., Zhang H. (2024). Integrating machine learning and multi-omics to identify key SUMOylation molecular signature in sarcoma. Life Conflux.

[9] Cheng Y., Kou W., Zhu D., Yu X., Zhu Y. (2021). Future directions in diagnosis, prognosis and disease monitoring of adrenocortical carcinoma: novel non-invasive biomarkers. Front Endocrinol (Lausanne).

[10] Custodio G., Komechen H., Figueiredo F.R., Fachin N.D., Pianovski M.A., Figueiredo B.C. (2012). Molecular epidemiology of adrenocortical tumors in southern Brazil. Mol Cell Endocrinol.

[11] Fang G., Yu H., Kirschner M.W. (1998). Direct binding of CDC20 protein family members activates the anaphase-promoting complex in mitosis and G1. Mol Cell.

[12] Morgan D.O. (1999). Regulation of the APC and the exit from mitosis. Nat Cell Biol.

[13] Peters J.M. (2002). The anaphase-promoting complex: proteolysis in mitosis and beyond. Mol Cell.

[14] Choi J.W., Kim Y., Lee J.H., Kim Y.S. (2013). High expression of spindle assembly checkpoint proteins CDC20 and MAD2 is associated with poor prognosis in urothelial bladder cancer. Virchows Arch.

[15] Kato T., Daigo Y., Aragaki M., Ishikawa K., Sato M., Kaji M. (2012). Overexpression of CDC20 predicts poor prognosis in primary non-small cell lung cancer patients. J Surg Oncol.

[16] Ding Z.Y., Wu H.R., Zhang J.M., Huang G.R., Ji D.D. (2014). Expression characteristics of CDC20 in gastric cancer and its correlation with poor prognosis. Int J Clin Exp Pathol.

[17] Wu W.J., Hu K.S., Wang D.S., Zeng Z.L., Zhang D.S., Chen D.L. (2013). CDC20 overexpression predicts a poor prognosis for patients with colorectal cancer. J Transl Med.

[18] Giordano T.J., Kuick R., Else T., Gauger P.G., Vinco M. (2009). Bauersfeld J.et al. Molecular classification and prognostication of adrenocortical tumors by transcriptome profiling. Clin Cancer Res.

[19] Gautier L., Cope L., Bolstad B.M., Irizarry R.A. (2004). affy–analysis of Affymetrix GeneChip data at the probe level. Bioinformatics.

[20] Pinto E.M., Rodriguez-Galindo C., Choi J.K., Pounds S., Liu Z., Neale G. (2016). Prognostic Significance of Major Histocompatibility Complex Class II Expression in Pediatric Adrenocortical Tumors: A St. Jude and Children’s Oncology Group Study. Clin Cancer Res.

[21] Leek J.T., Johnson W.E., Parker H.S., Jaffe A.E., Storey J.D. (2012). The sva package for removing batch effects and other unwanted variation in high-throughput experiments. Bioinformatics.

[22] Ritchie M.E., Phipson B., Wu D., Hu Y., Law C.W., Shi W. (2015). limma powers differential expression analyses for RNA-sequencing and microarray studies. Nucleic Acids Res.

[23] Wu T., Hu E., Xu S., Chen M., Guo P., Dai Z. (2021). clusterProfiler 4.0: a universal enrichment tool for interpreting omics data. Innovation (Camb).

[24] Groeneveld C.S., Chagas V.S., Jones S.J.M., Robertson A.G., Ponder B.A.J., Meyer K.B. (2019). RTNsurvival: an R/Bioconductor package for regulatory network survival analysis. Bioinformatics (Oxford, England).

[25] Blanche P., Dartigues J.-F., Jacqmin-Gadda H. (2013). Estimating and comparing time-dependent areas under receiver operating characteristic curves for censored event times with competing risks. Stat Med.

[26] Zhong C., Long Z., Yang T., Wang S., Zhong W., Hu F. (2023). M6A-modified circRBM33 promotes prostate cancer progression via PDHA1-mediated mitochondrial respiration regulation and presents a potential target for ARSI therapy. Int J Biol Sci.

[27] Zeng D., Ye Z., Shen R., Yu G., Wu J., Xiong Y. (2021). IOBR: multi-omics immuno-oncology biological research to decode tumor microenvironment and signatures. Front Immunol.

[28] Jiang P., Gu S., Pan D., Fu J., Sahu A., Hu X. (2018). Signatures of T cell dysfunction and exclusion predict cancer immunotherapy response. Nat Med.

[29] Hoshida Y., Brunet J.P., Tamayo P., Golub T.R., Mesirov J.P. (2007). Subclass mapping: identifying common subtypes in independent disease data sets. PLoS One.

[30] Hugo W., Zaretsky J.M., Sun L., Song C., Moreno B.H., Hu-Lieskovan S. (2017). Genomic and transcriptomic features of response to anti-PD-1 therapy in metastatic melanoma. Cell.

[31] Riaz N., Havel J.J., Makarov V., Desrichard A., Urba W.J., Sims J.S. (2017). Tumor and microenvironment evolution during immunotherapy with nivolumab. Cell.

[32] Braun D.A., Hou Y., Bakouny Z., Ficial M., Sant' angelo M., Forman J. (2020). Interplay of somatic alterations and immune infiltration modulates response to PD-1 blockade in advanced clear cell renal cell carcinoma. Nat Med.

[33] Zhao J., Chen A.X., Gartrell R.D., Silverman A.M., Aparicio L., Chu T. (2019). Author Correction: Immune and genomic correlates of response to anti-PD-1 immunotherapy in glioblastoma. Nat Med.

[34] Mayakonda A., Lin D.-C., Assenov Y., Plass C., Koeffler H.P. (2018). Maftools: efficient and comprehensive analysis of somatic variants in cancer. Genome Res.

[35] Gu Z., Eils R., Schlesner M. (2016). Complex heatmaps reveal patterns and correlations in multidimensional genomic data. Bioinformatics.

[36] Shen W., Song Z., Zhong X., Huang M., Shen D., Gao P. (2022). Sangerbox: a comprehensive, interaction-friendly clinical bioinformatics analysis platform. iMeta.

[37] Libé R., Huillard O. (2023). Adrenocortical carcinoma: Diagnosis, prognostic classification and treatment of localized and advanced disease. Cancer Treat Res Commun.

[38] Mir M.C., Klink J.C., Guillotreau J., Long J.-A., Miocinovic R., Kaouk J.H. (2013). Comparative outcomes of laparoscopic and open adrenalectomy for adrenocortical carcinoma: single, high-volume center experience. Ann Surg Oncol.

[39] Rodionov V., Nadezhdina E., Borisy G. (1999). Centrosomal control of microtubule dynamics. Proc Natl Acad Sci U S A.

[40] de Forges H., Bouissou A., Perez F. (2012). Interplay between microtubule dynamics and intracellular organization. Int J Biochem Cell Biol.

[41] Etienne-Manneville S. (2013). Microtubules in cell migration. Annu Rev Cell Dev Biol.

[42] Karamanos N.K., Theocharis A.D., Piperigkou Z., Manou D., Passi A., Skandalis S.S. (2021). A guide to the composition and functions of the extracellular matrix. FEBS J.

[43] Mondal G., Sengupta S., Panda C.K., Gollin S.M., Saunders W.S., Roychoudhury S. (2007). Overexpression of Cdc20 leads to impairment of the spindle assembly checkpoint and aneuploidization in oral cancer. Carcinogenesis.

[44] Kim Y., Choi J.-W., Lee J.-H., Kim Y.-S. (2019). Spindle assembly checkpoint MAD2 and CDC20 overexpressions and cell-in-cell formation in gastric cancer and its precursor lesions. Hum Pathol.

[45] Zhang X., Zhang X., Li X., Bao H., Li G., Li N. (2021). Connection between CDC20 expression and hepatocellular carcinoma prognosis. Med Sci Monit.

[46] Shi J., Chen Y., Gu X., Wang X., Liu J., Chen X. (2022). The prognostic assessment of CDC20 in patients with renal clear cell carcinoma and its relationship with body immunity. Contrast Media Mol Imaging.

[47] Song C., Kendi A.T., Lowe V.J., Lee S. (2022). The A20/TNFAIP3-CDC20-CASP1 axis promotes inflammation-mediated metastatic disease in triple-negative breast cancer. Anticancer Res.

[48] Chang D.Z., Ma Y., Ji B., Liu Y., Hwu P., Abbruzzese J.L. (2012). Increased CDC20 expression is associated with pancreatic ductal adenocarcinoma differentiation and progression. J Hematol Oncol.

[49] Becht E., Giraldo N.A., Dieu-Nosjean M.-C., Sautès-Fridman C., Fridman W.H. (2016). Cancer immune contexture and immunotherapy. Curr Opin Immunol.

[50] Chen D.S., Mellman I. (2013). Oncology meets immunology: the cancer-immunity cycle. Immunity.

[51] Wu F., Wang M., Zhong T., Xiao C., Chen X., Huang Y. (2023). Inhibition of CDC20 potentiates anti-tumor immunity through facilitating GSDME-mediated pyroptosis in prostate cancer. Exp Hematol Oncol.

[52] Rahim M.K., Okholm T.L.H., Jones K.B., McCarthy E.E., Liu C.C., Yee J.L. (2023). Dynamic CD8+ T cell responses to cancer immunotherapy in human regional lymph nodes are disrupted in metastatic lymph nodes. Cell.

[53] Strong L.C., Williams W.R., Tainsky M.A. (1992). The Li-Fraumeni syndrome: from clinical epidemiology to molecular genetics. Am J Epidemiol.

[54] Birch J.M., Blair V., Kelsey A.M., Evans D.G., Harris M., Tricker K.J. (1998). Cancer phenotype correlates with constitutional TP53 genotype in families with the Li-Fraumeni syndrome. Oncogene.

[55] Zhao S., Zhang Y., Lu X., Ding H., Han B., Song X. (2021). CDC20 regulates the cell proliferation and radiosensitivity of P53 mutant HCC cells through the Bcl-2/Bax pathway. Int J Biol Sci.

[56] Chukkalore D., Macdougall K., Master V., Bilen M.A., Nazha B. (2024). Adrenocortical carcinomas: molecular pathogenesis, treatment options, and emerging immunotherapy and targeted therapy approaches. Oncologist.

[57] Stephens D.M., Byrd J.C. (2012). Chronic lymphocytic leukemia with del(17p13.1): a distinct clinical subtype requiring novel treatment approaches. Oncology (Williston Park).

[58] Hassan N., Zhao J.T., Glover A., Robinson B.G., Sidhu S.B. (2019). Reciprocal interplay of miR-497 and MALAT1 promotes tumourigenesis of adrenocortical cancer. Endocr Relat Cancer.

[59] Litviakov N.V., Cherdyntseva N.V., Tsyganov M.M., Slonimskaya E.M., Ibragimova M.K., Kazantseva P.V. (2016). Deletions of multidrug resistance gene loci in breast cancer leads to the down-regulation of its expression and predict tumor response to neoadjuvant chemotherapy. Oncotarget.

[60] Kim J.H., Dhanasekaran S.M., Mehra R., Tomlins S.A., Gu W., Yu J. (2007). Integrative analysis of genomic aberrations associated with prostate cancer progression. Cancer Res.

