# Supplementary material for: Cell division cycle 20 promotes tumor progression and predicts poor clinical outcome in childhood and adult adrenocortical carcinoma
Source: J Clin Transl Endocrinol. 2025 Jul 2;41:100406. doi: 10.1016/j.jcte.2025.100406 (PMC12272758; doi:10.1016/j.jcte.2025.100406)
Supplement: Supplementary Data 1 [file mmc1.pdf]

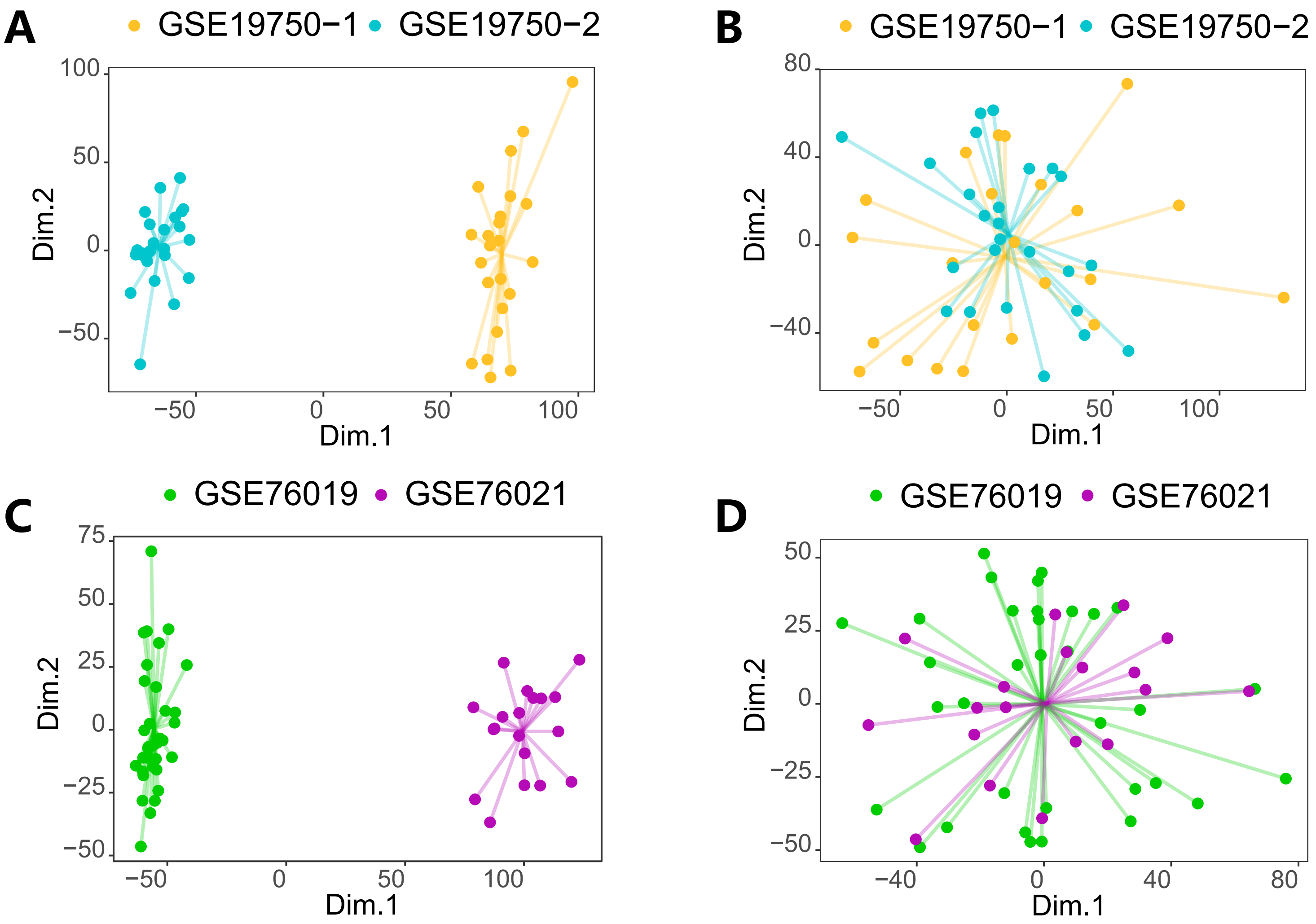

Figure S1: Batch correction of datasets. (A-B) Batch correction results of GSE76019 and GSE76021 datasets. (C-D) Internal batch correction results of GSE19750 dataset.

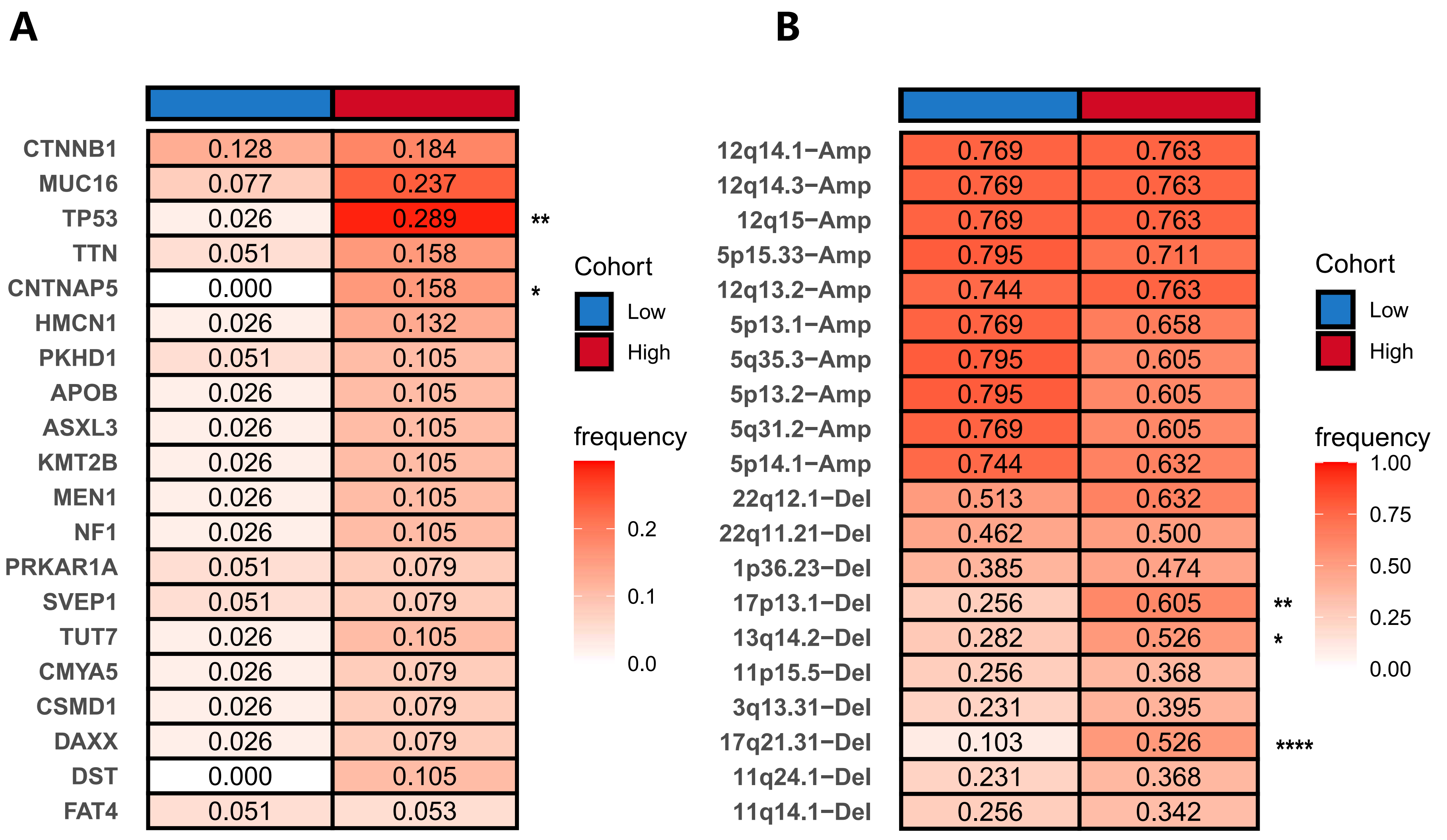

Figure S2: Differences in mutation frequency of genes and chromosomal fragment variation frequency between high and low CDC20 expression subgroups. (A) Mutation frequency of top 20 frequently mutated genes between high and low CDC20 expression groups. (B) The frequency of top 10 chromosomal amplifications and top 10 chromosomal deletions between CDC20 high-expression and low-expression groups. *P < 0.05, **P < 0.01, ***P < 0.001, ****P < 0.0001.
